# Appraisal of the technologies and review of the genomic landscape of ductal carcinoma in situ of the breast

**DOI:** 10.1186/s13058-015-0586-z

**Published:** 2015-06-16

**Authors:** Jia-Min B. Pang, Kylie L. Gorringe, Stephen Q. Wong, Alexander Dobrovic, Ian G. Campbell, Stephen B. Fox

**Affiliations:** Department of Pathology, Peter MacCallum Cancer Centre, St Andrews Place, East Melbourne, VIC 3002 Australia; Department of Pathology, University of Melbourne, Grattan Street, Parkville, Melbourne, VIC 3010 Australia; Sir Peter MacCallum Department of Oncology, University of Melbourne, Grattan Street, Parkville, Melbourne, VIC 3010 Australia; Cancer Genetics Laboratory, Peter MacCallum Cancer Centre, St Andrews Place, East Melbourne, VIC 3002 Australia; Translational Research Laboratory, Peter MacCallum Cancer Centre, St Andrews Place, East Melbourne, VIC 3002 Australia; Translational Genomics & Epigenomics Laboratory, Olivia Newton-John Cancer Research Institute, Studley Road, Heidelberg, VIC 3084 Australia

## Abstract

Ductal carcinoma in situ is a biologically diverse entity. Whereas some lesions are cured by local surgical excision, others recur as in situ disease or progress to invasive carcinoma with subsequent potential for metastatic spread. Reliable prognostic biomarkers are therefore desirable for appropriate clinical management but remain elusive. In common with invasive breast cancer, ductal carcinoma in situ exhibits many genomic changes, predominantly copy number alterations. Although studies have revealed the genomic heterogeneity within individual ductal carcinoma in situ lesions and the association of certain copy number alterations with nuclear grade, none of the genomic changes defined so far is consistently associated with invasive transformation or recurrence risk in pure ductal carcinoma in situ. This article will review the current landscape of genomic alterations in ductal carcinoma in situ and their potential as prognostic biomarkers together with the technologies used to define these.

## Introduction

Ductal carcinoma in situ (DCIS), the direct precursor to invasive carcinoma of the breast, is a clinical challenge. Thirteen to thirty-five percent of DCIS will recur within 10 years after local surgical excision [1]. These recurrence rates can be significantly reduced with adjuvant treatments, including localised radiotherapy [1, 2] and endocrine therapy [2, 3], but as there is no reliable way of identifying these cases, many patients are either under- or over-treated, leading to concomitant morbidity and cost [4]. Therefore, identifying DCIS cases with intrinsically aggressive behaviour is essential for appropriate allocation of any adjuvant treatment at the time of diagnosis.

Clinical and histopathological features associated with increased risk of ipsilateral recurrence include young patient age, symptomatic tumour detection, tumour multifocality, large tumour size, involved surgical margins, high nuclear grade, and presence of comedo necrosis [5]. In addition, protein biomarkers [6] and multigene expression assays such as the Onco*type* DX DCIS score [7] have shown potential as predictive and prognostic markers in selected patients.

Breast cancer not only is driven by somatic point mutations and epigenetic alterations but also characterised by extensive copy number changes [8, 9], and these large-scale alterations are likely to be informative of its biology in addition to clinico-histopathological features and expression profiles. However, little is known about the specific genetic alterations that drive an in situ malignancy to recur after local excision or progress to invasion.

Assessing the impact of genetic alterations on DCIS outcome is difficult. Cases of pure DCIS (DCIS in the absence of synchronous invasive carcinoma) have been infrequent until the widespread implementation of mammographic screening programmes; thus, establishing large cohorts of cases with long-term clinical follow-up presents a challenge. Such cohorts are required as the incidence of DCIS recurrence or invasive progression is relatively low and may occur many years after the initial DCIS episode. In addition, DCIS often appears as small lesions that require microdissection for accurate sampling, and fresh frozen tissue is rarely available. This often limits genetic investigations to technologies compatible with small quantities of formalin-fixed paraffin-embedded (FFPE)-derived DNA that is highly fragmented, especially in older samples [10–14]. In this review, we describe the key genomic alterations associated with DCIS, focus on their association with clinical and histological variables, and highlight the challenges in translating them for predicting prognosis and treatment strategies.

## Copy number changes in ductal carcinoma in situ

### Detection methods

Commonly used methods to detect genomic alterations have been comprehensively reviewed elsewhere [15], and methods frequently used in DCIS studies are summarized in Table 1 [16–22]. These methods can generally be divided into genome-wide or locus-specific approaches. Genome-wide approaches used in studying DCIS include chromosomal comparative genomic hybridisation (CGH), array comparative genomic hybridisation (aCGH), single-nucleotide polymorphism (SNP)-based arrays, and massively parallel sequencing (MPS).

**Table 1:** Common methodologies used for copy number variation detection

Method	Brief outline of method	Advantages	Disadvantages	Resolution	Main use
Chromosome comparative genomic hybridisation (CGH)	Target DNA and normal reference DNA differentially labelled and applied to metaphase spread from cultured normal lymphocytes	Genome-wide analysis	Cannot detect balanced chromosomal alterations or polyploidy. Resolution limited by use of highly condensed metaphase chromosomes	High-level amplification 250 kb Gains 2 Mb Losses 10 to 20 Mb [16]	Discovery studies
Array CGH (aCGH)	Target DNA hybridised to DNA clones (for example, bacterial artificial chromosomes) or oligonucleotides placed at certain intervals through genome.	Genome-wide analysis	Cannot detect balanced chromosomal alterations or polyploidy. Prone to spatial bias.	Determined by density of clone coverage	Discovery studies
Single-nucleotide polymorphism (SNP) arrays	Target DNA hybridised to oligonucleotides specific to SNPs and compared with collection of controls	Can detect loss of heterozygosity (LOH) and mutations. Normal reference DNA not required.	May not be genome-wide analysis as SNPs are unevenly distributed across genome; however, commercially available arrays deliberately include probes in SNP-poor areas to increase genome coverage. Prone to spatial bias.	Determined by length, density, and distribution of probes	Discovery studies
Molecular inversion probe array	Target DNA amplified in SNP-dependent manner and hybridised to oligonucleotides	Suitable for small amounts (<100 ng) of degraded DNA. Can detect LOH and mutations.	As for SNP arrays	Determined by density and distribution of probes	Discovery studies
Massively parallel sequencing	Parallel sequencing of large numbers (potentially millions) of templates	Potential genome-wide analysis. Can identify copy number neutral structural variations. Suitable for fragmented DNA.	Large volume of sequencing and data analysis	Potential single-base resolution	Discovery studies
Fluorescence in situ hybridisation	Fluorescently labelled genomic clones hybridised to target interphase nuclei	Structural rearrangements and polyploidy can be detected.	Minimal multiplexing ability	50 kb [17]	Locus-specific copy number analysis
Quantitative polymerase chain reaction (PCR)	Quantitation of copy number based on rate of amplification	Low DNA input requirements	Limited multiplexing ability. Prone to PCR amplification bias. Precision dependent on number of replicates. Underestimates high copy numbers.	Assay design dependent, but resolution of less than 100 base pairs (bp) possible.	Locus-specific copy number analysis
Droplet digital PCR	Quantification of copy number based on Poisson distribution statistics of thousands of digital PCRs [18]	Low DNA input requirements and compatible with fragmented DNA	Minimal multiplexing ability. Cannot detect polyploidy.	Targets regions of less than 100 bp possible. Can detect more than 0.15 % positive droplets per sample [19].	Locus-specific copy number analysis
Multiplex amplification and probe hybridisation (MAPH)/multiplex ligation-dependent probe amplification	Quantification of PCR products of hybridised probes	Multiplexable	Large amount of good-quality DNA required for MAPH (250 to 1,000 ng, >100 bp) [20]	150 bp [21, 22]	Locus-specific copy number analysis
Nanostring nCounter system	Absolute quantification of probes hybridised to target region	Multiplexable. Requires fragments of 100 bp or greater	Requires 300 ng of input DNA	Detects 0 to 4 copies of minimum 100 bp target regions	Locus-specific copy number analysis

CGH [23] involves the hybridisation of labelled target DNA to metaphase spreads along with differently labelled, normal, reference DNA. The contribution of CGH to understanding of genomic changes in breast cancer and precursor lesions has been reviewed by Reis-Filho and colleagues [24] (2005). aCGH, which has improved resolution and sensitivity compared with CGH in detecting copy number alterations, involves the hybridisation of target DNA to an array of DNA clones, often bacterial artificial chromosomes (BACs) spread at predetermined intervals along the genome. Further improvements in resolution have been developed by using SNP-based arrays, which also have the ability to detect loss of heterozygosity (LOH) and allelic imbalance. Copy number assessment by SNP arrays involves hybridising target DNA to oligonucleotides specific to SNPs. The data are compared with an independently hybridised group of controls instead of direct comparison with a presumed normal sample. These methods are generally not ideal for use with FFPE-derived DNA, and none is able to detect balanced chromosomal alterations or genomic polyploidy.

Molecular inversion probe (MIP) arrays were initially developed for SNP genotyping but also can be used to detect copy number alterations, LOH, insertions and deletions, and somatic mutations. The basis of this technique is the padlock probe which hybridises to either side of the target SNP. After enrichment for the closed probes, the probes are cleaved, amplified, and hybridised to an array. This approach is particularly suitable for DCIS samples as MIP arrays are compatible with small amounts (75 ng) of fragmented DNA [25].

A new technique which can overcome many of the limitations of array-based methods is MPS. MPS allows the simultaneous detection of genomic events at multiple loci in a high-throughput manner and not only can identify point mutations but can provide accurate copy number and, in the case of whole-genome sequencing, chromosomal translocation information as well. This approach has facilitated the exploration of the cancer genome of many tumours, including collaborative efforts such as The Cancer Genome Atlas (TCGA) programme, as well as the identification of clinically relevant alterations in diagnostic material [26, 27].

Whole-exome sequencing has been performed on a small number of DCIS samples [28]; however, these are unusual for DCIS as they are derived from fresh frozen tissue, requiring DNA amounts that are unachievable for most DCIS samples. Targeted MPS panels designed for FFPE-derived DNA, such as the TruSeq Amplicon Cancer Panel, have also been performed but again are biased against samples with extensive DNA degradation as these assays require fragment sizes of at least 170 base pairs (bp). These sequencing-based methodologies present additional challenges as FFPE-derived samples are acknowledged to give rise to sequencing artefacts, complicating data analysis [12, 29]. In addition, relatively small panels like this can detect alterations at the included target loci only.

Locus-specific copy number assays mainly have a role in detecting known copy number alterations and validating results of genome-wide copy number analyses. Earlier studies employed microsatellite markers to determine allelic imbalance and LOH. Locus-specific methods currently in widespread use include: quantitative polymerase chain reaction (qPCR), which quantifies copy number on the basis of rate of amplification; droplet digital PCR (ddPCR), which deduces copy number on the basis of limiting dilution involving thousands of individual PCRs; and fluorescence in situ hybridisation (FISH). The advantage of FISH is the ability to detect balanced structural rearrangements and polypoidy and to target a specific tissue area for copy number analysis without microdissection.

In addition, the recently developed Nanostring nCounter system allows probes targeting up to 800 regions of interest to be multiplexed and accurately counts barcoded probes hybridised to the target region to give a count of template copy number. This system is reported to require 300 ng of DNA and to be suitable for degraded FFPE material because of the relatively short (100 bp) probes used.

### Copy number alterations and nuclear grade

The most studied association of copy number alterations to DCIS phenotype is with nuclear grade. Most of these studies were performed in the 1990s and early 2000s by using CGH, which now is considered a low-resolution technique but at the time was a major advance in the detection of copy number alterations throughout the whole genome.

These studies demonstrated high levels of genomic instability in high-nuclear grade DCIS whereas low-nuclear grade DCIS showed fewer genomic alterations [24, 30–35]. In addition to general levels of genomic instability, high-grade and low-grade DCIS are distinguished from each other by recurrent chromosomal changes. These chromosomal changes are similar to those observed in grade 3 and grade 1 invasive carcinomas of the breast, respectively. Thus, low-grade DCIS is characterized by frequent 16q loss [31, 36–39] and 1q gain [31, 33, 36, 40], whereas high-grade DCIS shows frequent gain of 5p, 8q, 17q, and 20q [32, 33, 36–38], amplifications of 11q13, 17q12, and 17q22-24 [31, 32, 36], and loss of 8p, 11q, 13q, and 14q [31, 38, 39, 41]. The pattern of 8p loss has also been reported to differ between high-grade DCIS, in which whole arm loss (65 %) mostly occurs, and low- and intermediate-grade pure DCIS, in which 8p loss occurs as partial arm loss combined with proximal gain (29 %) rather than as whole arm loss (12 %) [38]. In addition to copy number changes, LOH of chromosome 17 [36] and regions 6q25-q27, 8q24, 9p21, 13q14, and 17p13.1 are more frequently reported in poorly differentiated DCIS [34], whereas LOH of chromosome 16 [36] and 16q22.3-q24.3 were more frequently altered in low-grade DCIS [34].

Whereas high-grade and low-grade DCIS are separated by different chromosomal alterations, intermediate-grade DCIS harbours changes overlapping with high- and low-grade DCIS [24, 31, 37]. This feature may be a consequence of the known poor reproducibility of intermediate-nuclear grade assignment by histopathologists [42] but also may reflect the biology of intermediate-grade DCIS. Gene expression profiling in invasive breast cancers has revealed that histologically grade 2 breast cancers do not have a distinct gene expression pattern but that instead many of these tumours have expression profiles similar to those of histologically grade 1 or grade 3 tumours, which were associated with low and high risk of recurrence, respectively [43]. Given the parallels between DCIS and invasive carcinoma, it could be expected that intermediate-grade DCIS similarly is not a distinct independent entity but encompasses cases that align with low-grade DCIS and high-grade DCIS. This raises the issue of how intermediate-nuclear grade cases should be classified for the purposes of genomic studies and whether the common practice of merging intermediate-grade cases with low-grade cases to create a ‘non-high grade’ group is a valid approach when some of the intermediate-grade DCIS cases may be biologically ‘high grade’.

**Fig. 1:**
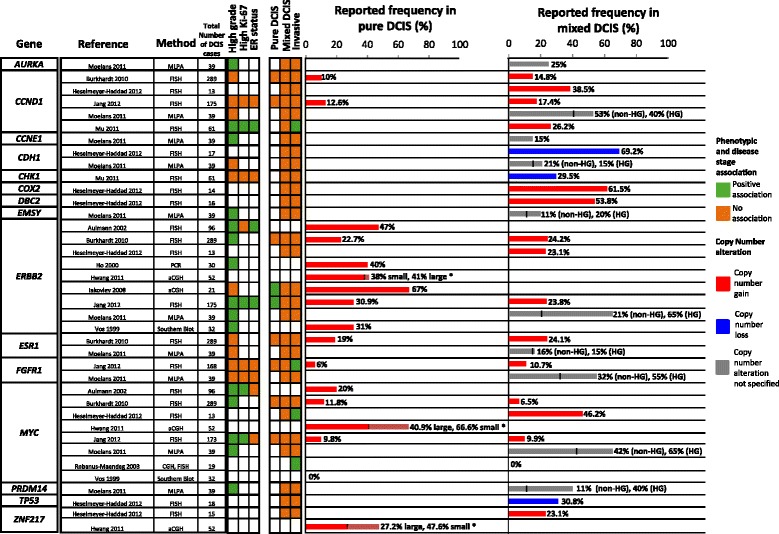
Copy number alterations of specific genes associated with ductal carcinoma in situ (DCIS) phenotype and disease stage. *Small DCIS lesions are defined as less than 15 mm, and large DCIS lesions are defined as more than 40 mm. aCGH, array-comparative genomic hybridization; CGH, comparative genomic hybridization; ER, estrogen receptor; FISH, fluorescence in situ hybridisation; HG, high grade; MLPA, multiplex ligation-dependent probe amplification; non-HG, non-high grade; PCR, polymerase chain reaction

In terms of individual genes, *MYC* (8q24) and *ERBB2* (17q12) amplifications have been associated with high nuclear grade [44–47] and with other features suggestive of a more aggressive phenotype such as high Ki-67 index [45, 46] and micropapillary and comedo growth patterns, respectively [45]. Copy number alterations of *CCNE1* (19q12) and *AURKA* (20q13) have been reported to occur exclusively in high-grade DCIS [47], while the 11q13 amplicon, frequently present in high-grade DCIS, contains the known oncogene *CCND1* [32] (Fig. 1).

### Copy number alterations and intrinsic subtype

In invasive breast cancer, the intrinsic subtypes of luminal A, luminal B, HER2, and triple-negative were defined by gene expression profiling [48, 49] and were found to correlate with survival and copy number profiles [50]. These subtypes can be approximated by surrogate immunohistochemical markers [51–53]. Vincent-Salomon and colleagues (2008) [54] identified different patterns of copy number alterations by intrinsic subtype in 57 cases of pure DCIS. In the *HER2*-amplified DCIS subgroup, half of recurrent amplicons other than *HER2* (13 out of 26, 50 %) were also on chromosome 17q, as opposed to just five of 29 amplicons (17.2 %) in luminal-subtype DCIS [54]. This study identified specific regions of gain (17q) and losses (3p, 4p, 4q, and 8p) in *HER2*-amplified DCIS and specific regions of gain (1q, 8p, and 17q) and loss (16q) in luminal-subtype DCIS [54]. They also classified DCIS cases into categories based on the type and degree of copy number alterations, similar to that proposed in invasive carcinomas [55]. Tumours were characterized by (i) few copy number changes apart from 1q gain and 16q loss (classified as ‘1q/16q’), (ii) tumours with many low-level copy number alterations (labelled ‘complex’), and (iii) tumours with recurrent amplifications in addition to low-level copy number alterations (‘mixed amplifiers’) [55, 56]. This study provides a novel, though not yet clinically validated, molecular classification system for DCIS.

### Copy number alterations of chromosomal regions: comparisons between pure DCIS, mixed DCIS, and invasive carcinoma

Paradoxically, several studies have reported greater genomic instability in pure DCIS compared with mixed DCIS (DCIS adjacent to invasive carcinoma) [40, 57]. Farabegoli and colleagues [57] (2002), using 15 microsatellite markers in regions altered in invasive breast cancer, reported more frequent LOH in 28 pure DCIS lesions compared with 25 mixed DCIS cases. More recently, Liao and colleagues [40] (2012) used 19K BAC arrays followed by qPCR validation to investigate copy number alterations in 20 low-grade pure DCIS, 25 low-grade mixed DCIS, and 24 of their matched invasive components. Pure DCIS harboured more copy number alterations compared with mixed DCIS. Sixteen regions on 13 chromosomal arms had a statistically significant difference in copy number alterations between pure DCIS and mixed DCIS, all but one of which were increased copy number gains in pure DCIS [40]. The authors hypothesise that the copy number gains in low-grade pure DCIS lesions may result in amplification and possible upregulation of invasion suppressor genes [40]. However, given that many of the alterations in low-grade DCIS are in common with invasive carcinoma, this seems unlikely. Alternatively, the high genomic instability in pure DCIS may not allow the cohesive and sustained signalling of pro-invasive pathways for invasion to occur. Furthermore, the results may be biased by the presence of normal cells (for example, lymphocytes) within regions of invasive tumour that are more readily avoided when microdissecting pure DCIS because of the contained nature of the lesion. Thus, reduced detection sensitivity may be a technical reason for lower levels of copy number alterations in invasive breast cancer compared with DCIS.

It is interesting, but perhaps not surprising, to note that in contrast to comparisons with pure DCIS, the majority of DCIS cases with associated invasive carcinoma show genomic changes that are remarkably concordant with their matched invasive component [31, 35, 38, 40, 41, 47, 58–61]. These studies have examined up to 24 synchronous DCIS-invasive carcinoma pairs and found identical chromosomal alterations in at least 75 % of cases [31, 35, 38, 40, 41, 58, 61]. Johnson and colleagues [35] (2012) investigated copy number differences between the DCIS and invasive carcinoma components of 21 tumours by using MIP arrays, providing the greatest resolution of the copy number differences between matched DCIS and invasive carcinoma to date. Shared copy number alterations were present in 81 % (17 out of 21) of pairs. In addition, exclusive regions of copy number gain (5q, 16p, 19q, and 20) and copy number loss (3q, 6q, 8p, and 11q) were also identified in the invasive component as well as a region of exclusive copy number loss (17q11.2) in DCIS [35].

Although genomic similarities between synchronous DCIS and invasive carcinoma are likely to indicate direct development of invasive carcinoma from the DCIS component, these findings could also potentially arise from ductal colonisation by invasive carcinoma, mimicking DCIS. Similarly, genomic differences between DCIS and invasive carcinoma components may indicate genetic changes important in determining invasion but could also be due to clonal heterogeneity and ongoing genetic evolution.

### Copy number alterations of specific genes: comparisons between pure DCIS, mixed DCIS, and invasive carcinoma

Copy number alterations of specific genes reported to be associated with disease progression include those whose protein products regulate the cell cycle (*CCND1*) or transcription (*MYC*) or function as receptor tyrosine kinases (*ERBB2* and *FGFR1*) (Fig. 1). Many of these (for example, *FGFR1*) are also targets of amplification in other cancers.

When pure DCIS and mixed DCIS are compared, one study has reported a number of genes showing different frequency of copy number gains and losses [40]. However, apart from *SMRT* (*NCOR2*) and *NR4A1* (both on 12q24 and showing increased gain in pure DCIS and increased loss in mixed DCIS), none of the genes has been associated with breast cancer in other studies. In addition, application of these results to the general DCIS population may not be appropriate as the study population was limited to low-grade cases [40]. Another study involving a cohort of 130 pure DCIS and 159 mixed DCIS found no difference in copy number of the breast cancer- related genes *ERBB2*, *ESR1*, *CCND1*, and *MYC* by FISH between the two types of DCIS [60].

Comparing DCIS and invasive components of synchronous tumours, Johnson and colleagues [35] (2012) observed increased amplitude of copy number gain in the invasive component compared with matched DCIS in regions encompassing known oncogenes *MYC* and *CCND1*. This observation is in keeping with an earlier report of an increase in amplitude of *MYC* amplification in invasive carcinoma compared with adjacent DCIS [62] and a recent study which reported *MYC* amplification to be present in more than 30 % of tumour cells in 10 out of 13 (76.0 %) invasive carcinoma cases compared with only six out of 13 (46.2 %) matched DCIS cases [63], but this was not confirmed in some other studies [46, 47, 60].

Mu and colleagues [64] (2011) also identified *CCND1* amplification in invasive carcinoma which was absent in matched adjacent DCIS in three of 16 (18.8 %) cases. Interestingly, in these three cases, the invasive components were high-grade but the adjacent DCIS were low-grade. These data indicate that *CCND1* may help mediate invasion and transition to a higher grade; however, it is also possible that in these cases the DCIS may not be a direct precursor of the invasive carcinoma. Significant differences in *CCND1* amplification between invasive carcinoma and matched DCIS were not detected in other studies [46, 63].

Although a previous smaller study (n = 39) revealed no difference in *FGFR1* copy number between mixed DCIS and matched invasive carcinoma [47], Jang and colleagues [46] (2012) examined a large cohort of pure DCIS (n = 175), mixed DCIS (n = 203), and invasive carcinoma (n = 427) by FISH and reported that *FGFR1* amplification (defined as average copy number of more than 6.0 or *FGFR1*-to-centromeric enumeration probe ratio of more than 2.2) was not only significantly more frequent in invasive carcinoma compared with pure DCIS (12.5 % versus 6.0 %, *P* = 0.020) but also more frequent in invasive carcinoma compared with matched adjacent DCIS (*P* = 0.031), suggesting a role of *FGFR1* amplification in the transition from non-invasive to invasive disease.

Whereas the studies highlighted above observed differences between the DCIS and invasive components of synchronous tumours, other studies have found no difference in copy number for the same and other genes [46, 47, 60, 63]. This may be due to the small number of cases included in some studies [47, 63] and the limited panel of genes selected for investigation. Alternatively, the similarities may be due to a direct clonal relationship between DCIS and adjacent invasive carcinoma and suggest that structural genome changes might not be responsible for the acquisition of an invasive phenotype.

### Heterogeneity of genomic changes in ductal carcinoma in situ

**Fig. 2:**
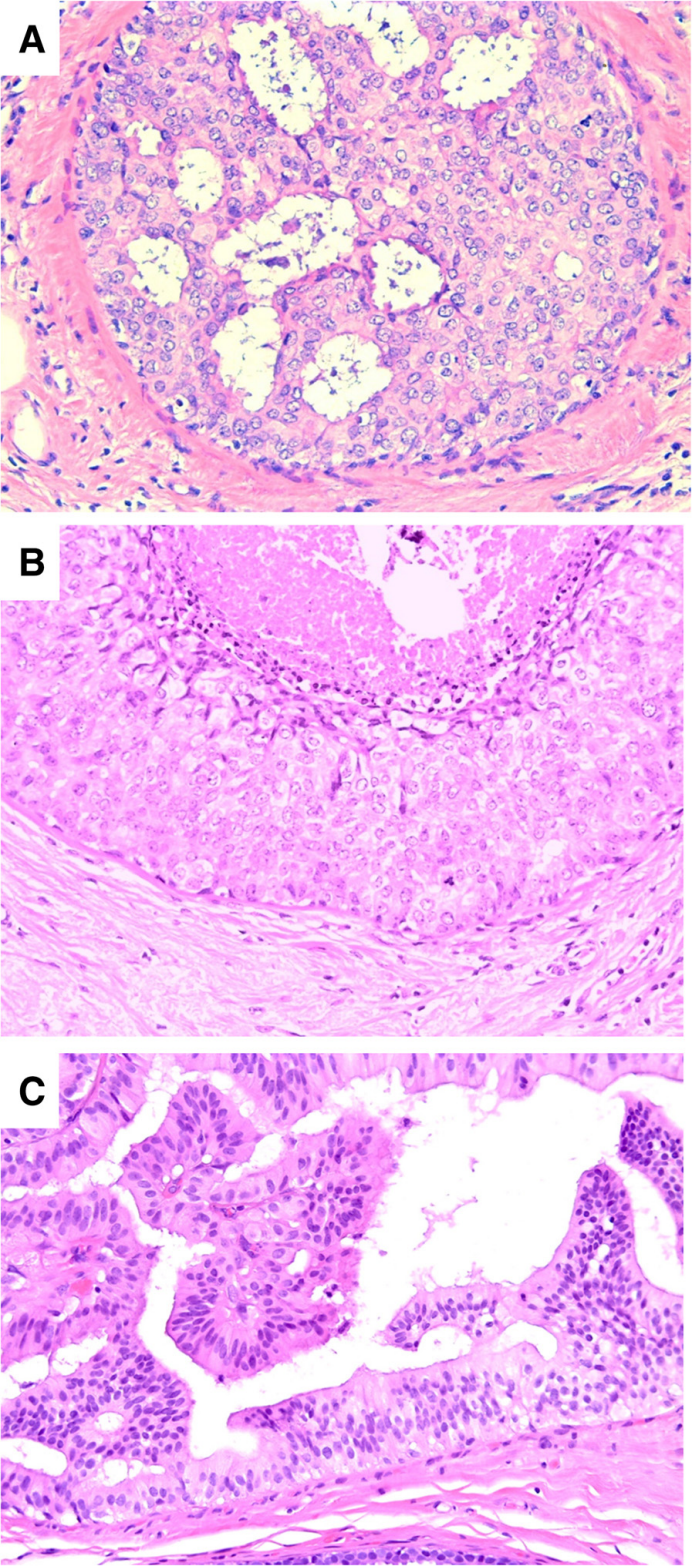
Single ductal carcinoma in situ (DCIS) lesion showing variation in nuclear grade and architectural patterns. (**a**) High-grade DCIS with cribriform architecture (200×). (**b**) High-grade DCIS with solid architecture with comedo necrosis (200×). (**c**) Low-grade DCIS with papillary and micropapillary architecture (200×)

**Fig. 3:**
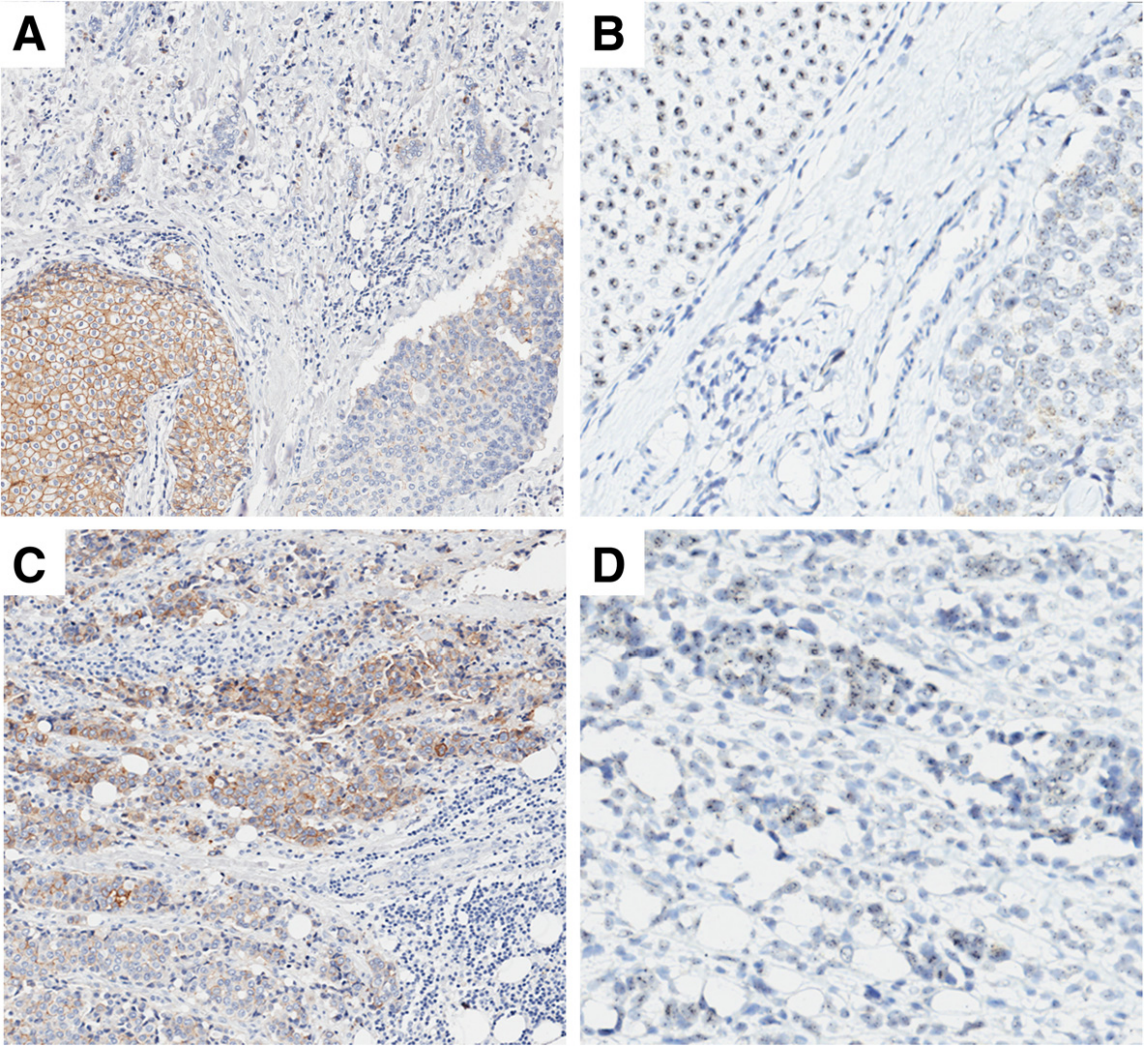
Tumour with synchronous ductal carcinoma in situ (DCIS) and invasive carcinoma showing heterogeneity of HER2 amplification and expression. (**a**) HER2 immunohistochemistry (IHC) showing strong circumferential membrane staining (positive for HER2) in an area of DCIS, with negative staining in adjacent DCIS and invasive carcinoma (100×). (**b**) *HER2* silver enhanced in situ hybridisation (SISH) of same region as (**a**) showing DCIS with *HER2* amplification and adjacent DCIS without *HER2* amplification (200×). (**c**) Invasive carcinoma with areas showing strong circumferential membrane staining for HER2 IHC (positive for HER2) and areas negative for HER2 (100×). (**d**) *HER2* SISH of same region as (**c**) showing invasive carcinoma with areas with and without *HER2* amplification (200×)

Studies examining copy number alterations in matched pairs of mixed DCIS and invasive carcinoma have also shed light onto the high degree of intra-tumoural genomic heterogeneity of DCIS. This result is unsurprising given the demonstrated morphological, immunohistochemical, and intrinsic subtype diversity within individual DCIS lesions [65] (Figs. 2 and 3). Hernandez and colleagues [61] (2012) identified both qualitative and quantitative differences in copy number between mixed DCIS and matched invasive carcinoma in three of 13 cases (23.1 %) by aCGH. Validation of the results by FISH revealed that the component showing lower copy numbers for the target region was in fact composed of a mosaic of cells, some of which harboured the amplification whereas some did not. Similarly, Jang and colleagues [46] (2012) observed discrepancies in *HER2*, *MYC*, *CCND1*, and *FGFR1* amplification between in situ and invasive components in 22 out of 203 matched pairs (10.8 %) by FISH on tissue microarrays. However, when FISH was performed on whole sections, heterogeneous amplification was observed and this may account for the discrepant findings.

Such data support the notion that DCIS lesions are genomically heterogeneous and undergo clonal selection as well as ongoing genetic evolution in the progression to invasive carcinoma. This idea is in keeping with the finding of greater diversity of allelic loss patterns in DCIS compared with invasive carcinomas [66]. Clonal divergence in the progression from DCIS to invasive carcinoma has been reported in a study involving FISH analysis of 13 matched DCIS-invasive carcinoma pairs by Heselmeyer-Haddad and colleagues [63] (2012). In this study, four patterns of clonal evolution were observed. Cases in the first category show the major clone to be unchanged in DCIS and invasive components. In the second category, one of several major clones in DCIS became the dominant clone in the invasive tumour, whereas in the third pattern, the major clone in DCIS became one of two major clones in the invasive tumour. The fourth pattern was characterized by a shift in the major clone between DCIS and invasive carcinoma [63]. However, in this study, the DCIS and invasive components were macrodissected to create cytospin preparations for FISH analysis, raising the possibility that contaminating non-tumour cells could be inadvertently included in the analysis because of loss of architectural features.

Nonetheless, the genetic diversity within DCIS lesions complicates the search for genomic changes which drive the transition to invasive phenotype. Perhaps, the mere presence of genetic diversity may itself be a marker of aggressive behaviour, similar to what has been reported for Barrett’s oesophagus [67], and is an avenue which warrants further investigation in DCIS.

Despite the utility of studying matched mixed DCIS-invasive carcinoma pairs, it can be argued that this approach is not appropriate for the identification of prognostic markers of pure DCIS and that studying differences between pure DCIS cases that do not recur and those that do is a better method of finding biomarkers that can be used to guide clinical management of patients who present with DCIS in the absence of synchronous invasive carcinoma. As yet, no studies have examined genomic differences between pure DCIS which are cured by local surgical excision and pure DCIS cases which recur as either in situ or invasive disease after local excision. One study did include 17 cases of pure DCIS with recurrences and 17 cases of pure DCIS without recurrence; however, no subgroup analysis was reported for these groups [38]. Another novel approach employed by the same group was to study a unique group of pure DCIS cases characterised by large tumour size and high nuclear grade but without evidence of invasion despite thorough histopathological examination [68]. These tumours were compared with small high-grade DCIS lesions to identify differences which may account for the lack of invasion in the large tumours. Large DCIS cases were found to have fewer copy number gains of *MYC* (40.9 % of large and 66.6 % of small DCIS) and of *ZNF217* (27.2 % of large and 47.6 % of small), and in cases with an above-median Ki-67 index, large DCIS lesions showed fewer amplifications compared with small lesions [68].

## Somatic mutations and rearrangements in ductal carcinoma in situ

Compared with copy number alterations, somatic mutations have been identified relatively infrequently in DCIS. One of the most frequently mutated genes in DCIS is *PIK3CA. PIK3CA* mutations are believed to be early events in the development of breast cancer [61, 69–71]. A higher frequency of *PIK3CA* mutations in mixed DCIS (8 out of 33, 24 %) than in pure DCIS (0 out of 31) has been reported in a specific group of high-grade, estrogen receptor-positive, HER2-negative DCIS cases [72]. However, Miron and colleagues [73] (2010) reported the frequency of *PIK3CA* activating mutations to be 30 % in pure DCIS (61 out of 202 cases) and mixed DCIS (29 out of 97 cases) and 29 % in invasive carcinoma (35 out of 120 cases). Of the matched mixed DCIS-invasive carcinoma cases, 25 % (19 out of 76) had discordant results between the two components, without any trend toward either group [73]. In addition, Johnson and colleagues [35] (2012) identified *PIK3CA* mutations in 8 out of 21 (38.1 %) matched mixed DCIS-invasive tumours; however, in two of these cases, the mutation was present in the DCIS component only. These findings suggest that *PIK3CA* mutations are not positively selected in the transition from in situ to invasive disease.

Although *AKT1* mutations are rare in invasive breast cancer (approximately 2 %) (TCGA), activating *AKT1* exon 2 mutations were observed in the in situ component of two of three breast tumours exhibiting the mutation in the invasive component in one study [69] and in three of six papillomas harbouring DCIS in another study [74]. Similar to invasive breast cancers [28], no coexistent *PIK3CA* mutations were detected in tumours with *AKT1* mutations consistent with their role within the same pathway [69].

*TP53* mutations in exons 4 to 11 have been reported in 10 % to 37 % of pure DCIS cases [44, 54, 75–79] and 20 % to 33 % of mixed DCIS cases [78, 79] and associated with high nuclear grade [54, 77] and HER2 subtype [54]. The presence of *TP53* mutations also appears to be an early event in breast cancer development and not specifically associated with in situ-to-invasive transition [79].

Translocations of two genes previously associated with increased oncogenic activity [80], *MAST2* and *NOTCH1*, were identified by FISH in one study in 4 out of 115 (3.5 %) and 2 out of 115 (1.7 %) mixed DCIS cases, respectively, but these translocations were not observed in 170 cases of pure DCIS [81]. However, structural rearrangements in DCIS may be more frequent than currently realized given the relatively frequent occurrence of such events in invasive breast cancers and breast cancer cell lines [82].

## Conclusions

Identifying patients curable by local surgical excision from those who have more aggressive biology and require additional treatment is important to spare low-risk patients from mastectomy and adjuvant treatments such as radiotherapy and hormonal blockade and their associated side effects and cost while preventing undertreatment of high-risk patients. Studies have identified multiple genomic changes and revealed the degree of intra-tumoural heterogeneity in DCIS. However, so far, none of these alterations is a reliable indicator of in situ recurrence or invasive progression.

The lack of a clear genomic signature of recurrence risk may be due to several reasons. Firstly, large cohorts of pure DCIS cases with long-term clinical follow-up are required to assess outcome in DCIS, and these have been rare to date. Data from randomised controlled trials investigating the utility of radiotherapy in DCIS indicate that 88 % of detected recurrences occur within 10 years. However, local recurrence events continue to occur after 10 years, suggesting that follow-up should extend beyond this period [1]. Secondly, global high-resolution genomic profiling using current technologies requires high-quality genomic material such as that derived from fresh tissue. Fresh DCIS tissue is very rarely available as the diagnosis of pure DCIS requires histological examination of the entire lesion. Therefore, collections of fresh-frozen DCIS tissue for research are unlikely to be established given diagnostic considerations. However, given the rapid development of technology in this field, it is likely that methodologies will evolve to be compatible with FFPE tissue before fresh-frozen DCIS tissue banks accrue sufficient cases. Currently, several methods are suitable for good-quality FFPE material, including MIP arrays and MPS, especially protocols using hybridisation-capture approaches, as well as more targeted methods such as the Nanostring nCounter system and ddPCR. It is envisioned that, in the future, technologies compatible with all FFPE tissues will be developed that can recover information of all aspects of the genome whether structural, copy number, or point mutations.

Thirdly, genomic alterations alone may not determine prognosis in DCIS. Instead, integration of genomic, epigenomic, and transcriptional data with clinico-histopathological features could be more informative of prognosis and lead to an improved understanding of the biology of DCIS, including the fundamental question of why local recurrences occur after apparently complete surgical excision. The role of myoepithelial cells and the tumour microenvironment in determining outcome in DCIS is increasingly being recognized [83, 84], and another possible explanation is the ‘sick lobe’ theory [85], which proposes that the entire lobe in which a lesion occurs is genetically unstable and prone to tumourigenesis, leading to the development of further tumours in the region. Genomic alterations in DCIS lesions could be informative of outcome by being markers of a certain relationship between the malignant cells and the tumour microenvironment permissive to local recurrence or by reflecting the genetic instability of the local breast field.

## Abbreviations

aCGH: array comparative genomic hybridization
BAC: Bacterial artificial chromosome
bp: base pairs
CGH: Comparative genomic hybridisation
DCIS: Ductal carcinoma in situ
ddPCR: droplet digital polymerase chain reaction
FFPE: Formalin-fixed paraffin-embedded
FISH: Fluorescence in situ hybridisation
LOH: Loss of heterozygosity
MIP: Molecular inversion probe
MPS: Massively parallel sequencing
qPCR: quantitative polymerase chain reaction
SNP: Single-nucleotide polymorphism
TCGA: The cancer genome Atlas
